# Avian Influenza Risk Surveillance in North America with Online Media

**DOI:** 10.1371/journal.pone.0165688

**Published:** 2016-11-23

**Authors:** Colin Robertson, Lauren Yee

**Affiliations:** Department of Geography & Environmental Studies, Wilfrid Laurier University, 75 University Ave West, Waterloo, ON, N2L 3C5, Canada; Public Health Agency of Canada, CANADA

## Abstract

The use of Internet-based sources of information for health surveillance applications has increased in recent years, as a greater share of social and media activity happens through online channels. The potential surveillance value in online sources of information about emergent health events include early warning, situational awareness, risk perception and evaluation of health messaging among others. The challenge in harnessing these sources of data is the vast number of potential sources to monitor and developing the tools to translate dynamic unstructured content into actionable information. In this paper we investigated the use of one social media outlet, Twitter, for surveillance of avian influenza risk in North America. We collected AI-related messages over a five-month period and compared these to official surveillance records of AI outbreaks. A fully automated data extraction and analysis pipeline was developed to acquire, structure, and analyze social media messages in an online context. Two methods of outbreak detection; a static threshold and a cumulative-sum dynamic threshold; based on a time series model of normal activity were evaluated for their ability to discern important time periods of AI-related messaging and media activity. Our findings show that peaks in activity were related to real-world events, with outbreaks in Nigeria, France and the USA receiving the most attention while those in China were less evident in the social media data. Topic models found themes related to specific AI events for the dynamic threshold method, while many for the static method were ambiguous. Further analyses of these data might focus on quantifying the bias in coverage and relation between outbreak characteristics and detectability in social media data. Finally, while the analyses here focused on broad themes and trends, there is likely additional value in developing methods for identifying low-frequency messages, operationalizing this methodology into a comprehensive system for visualizing patterns extracted from the Internet, and integrating these data with other sources of information such as wildlife, environment, and agricultural data.

## Introduction

Surveillance systems are essential to detect early warning signals in animal and human health and inform management strategies for populations at risk. As human populations continue to grow the demand for more resources from the environment, contact with animals increases. In turn this places pressures on wildlife populations, their habitat, and agricultural practices that are needed to meet the increasing demand for animal proteins globally. As these interfaces between wildlife, domestic animals and humans increase we can anticipate increased involvement with wildlife in emerging diseases [1].

The relationship between domestic livestock populations and wild reservoirs is important to understand in the context of disease transmission and evolution. Biosecurity measures on farms, mixed farming practices [2]open air farming [3] and animals in close proximity can all influence the emergence of disease. Clinical signs in live wild animals are difficult to observe. Typically, the impact on a population is more easily quantified for domestic animals than wild, and expressed in terms of economic losses, as their location and the population susceptible to disease is more often known. For example, the avian influenza A(H5N1) outbreaks in poultry during 2004–2009 caused an estimated $30 billion in damages [4]Zoonoses like avian influenza are particularly challenging to plan for and manage at local scales because of the complex inter-relationships between domestic and wild bird populations. In both economic and health terms, there is growing awareness of need for situational awareness surveillance tools to manage and adapt to variable, inter-connected disease landscapes [5]. More recently, researchers have been looking to create complimentary surveillance systems that utilize non-traditional forms of information. These new systems typically have the aim of analysing trends for early warning purposes, so that increased surveillance or action can take place [6] (However in the case of AI, we do not expect messages and media activity to provide early-warning of outbreaks, as it is likely that media reports are responding to official surveillance. In the parlance of Broniatowski [7], the signal obtained for social media for AI is likely dominated by ‘chatter’–not reports of new or unknown infection/ transmission events. However, from the perspective of public and animal health planning, there may be significant value in understanding how this chatter relates to real outbreaks of AI. We explore this question by investigating the extent to which statistical outbreaks of AI-related Twitter messages relate to true outbreaks reported in official surveillance data. We do this by developing an automated methodology to acquire, structure, and detect temporal outbreaks of AI-related Tweets, and then qualitatively investigating the topics and characteristics of messaging associated with detected outbreak periods. While we limit our analysis of risk surveillance to North America via Twitter, given the transboundary nature of AI transmission, we do not exclude outbreaks external to North America, as they remain relevant for understanding the global AI risk scenario from the North American perspective.

### Avian influenza

Avian influenza is a virus that can be transmitted from wild birds to domestic poultry. Some strains of avian influenza viruses (AIVs) can be asymptomatic or without clinical signs of illness in chickens, characterized as low pathogenic avian influenza viruses (LPAI), whereas other strains can cause severe disease that is fatal within a few days, these viruses are referred to as highly pathogenic avian influenza (HPAI) [8]In wild birds, AIV is generally asymptomatic [9]) but still poses a threat to public and animal health initiatives since wild birds can reach a wide geographic area [10]Of particular concern are waterfowl, which can cover large distances during migration, have the greatest subtype variety as well as having the highest general AIV prevalence rates [11]. These characteristics offer the opportunity for novel AIVs to emerge through co-infection events and introduce AIVs from different regions into immunologically naive populations [9]. Wild bird species are thought to transmit the virus by active shedding, mechanical transfer of water droplets or excretion of large amounts of the virus in wild bird feces [12]The high virus prevalence in water birds may be due to the transmission route through the fecal material in water [11]. The abundance of virus shed into lake water and wetlands can provide a way for ducks to spread the virus through migration onto other domestic or wild birds [11]In addition, introduction of LPAI viruses into a poultry population may not necessarily involve direct contact but rather the mechanical transfer of the virus through infective faeces from the waterfowl [13]. LPAI of subtypes H5 and H7 in wild birds can still be problematic as it can mutate when transmitted to poultry to the highly pathogenic type [14]. As was shown in February 2003 when an outbreak of pathogenic avian influenza (subtype H7N7) in the Netherlands is thought to have originated from free-living ducks that had evolved into a highly pathogenic variant after introduction into poultry farms [15].

Surveillance of wild birds and reporting of H5 and H7 subtypes of AIV by World Organization for Animal Health (OIE) member countries is a key component in the early detection of potential outbreaks at an international scale. Surveillance systems can be designed to meet a number of public health objectives such that each surveillance system may have different requirements in terms of data, methodology and implementation [16]. Objectives can include: description of disease trends over time (or disease status of population), detection of new disease events, and management. Passive surveillance obtains samples of sick or dead animals through routine activities by stakeholders (e.g. park wardens, hunters), while active surveillance is the direct action of searching for disease in animals through sampling; typically targeting specific geographic areas or populations [17].

A limiting factor of surveillance of wildlife is the difficulty in obtaining samples; often relying on targeted populations over localized areas, or the reporting of opportunistically found dead animals or live birds caught for other reasons. These samples could be from ornithologist-captured or hunter-collected birds [18]. Due to the inherent bias in samples of convenience, establishing valid estimates of disease absence or prevalence remains difficult [18]. HPAI detection has been traditionally based on passive surveillance since the infection induces clear clinical signs and high mortality in most poultry species [14]. In wild birds, dead bird collection limits the insights that can be made on the distribution, spread and the diversity of AIV strains, since death is rarely caused in wild birds [19]. In terms of early-warning, surveillance that catches events only after large mortality events occur could be improved by investigating auxiliary signals related to disease status (i.e., syndromic surveillance). Additionally, tracking outbreaks over global scales requires dealing with vast amounts of data, of varying quality and geographic and temporal precision.

Comin [14]used epidemiological data from Italian LPAI surveillance programs and simulated within-farm outbreaks using different surveillance strategies. They found that surveillance was only successful in preventing an epidemic in turkeys if action was taken within two days of sampling. Comin [14]further suggest that action within a two day window is unfeasible given the lag time that would be associated with diagnostic laboratory results. Hoye [18]further asserts that, “effective surveillance requires a compromise between sampling that is based on probability and the constraints of sample collection, transport and analysis, the details of which will depend on the specific objectives of the survey”. They suggest that using probability methods could be used to plan the species, locations and months of the year to sample, as well as utilizing birds sampled by hunters and ornithologists with additional samples taken to meet the probability thresholds required [18].

### Novel Surveillance Systems

Disease surveillance systems have traditionally relied upon data from hospitals or public health department records to monitor diseases across populations [20], or in the case of wildlife diseases and zoonoses, government departments of natural resources or agriculture, hunters, and sometimes individual researchers. As mentioned previously, action is often limited by the lag time between observed symptoms of disease (or active collection of samples), laboratory submission and results, and communication to the appropriate authorities. Novel approaches to surveillance aim to recognize patterns and provide timely indicators of a potential disease outbreak before they happen. There are a number of new data sources that can be utilized to predict, forecast and collect information for disease surveillance purposes. These approaches can be: sales of over-the-counter medicine, absences from work or school, patient’s chief complaint upon emergency visit, or laboratory test orders [21,22] and more recently, search term frequency for symptoms (e.g. [23,24]), web scraping (e.g. www.Healthmap.org) and social media (e.g. [25]).

For instance, Google uses search term frequency for influenzalike illness (ILI) in their system, termed Google Flu Trends (GFT) to estimate the number of physician visits in a particular city, state or region [26]. GFT has had mixed results, in Ginsberg [24] GFT was shown to predict weekly national ILI percentages 1–2 weeks ahead of the publication of the CDC’s US influenza Sentinel Provider Surveillance Network. GFT could then be used in the case of allocating additional personnel to emergency departments where emergency departments rely on coupling early detection with a graded rapid response to manage both seasonal and pandemic influenza surges [26]. However, it was found that GFT was reporting more than double the portion of doctor visits for ILI than the CDC and later missed the 2009 outbreak of influenza A-H1N1 [27]. The overestimating of flu prevalence as well as missing significant flu related events can be attributed to big data issues and poor choice of statistical methods and changes in algorithm dynamics (used to enhance google's search engine) [27]. Although GFT is promising in some use cases it is not yet at a level where it can replace traditional surveillance methods. The importance of sound statistical methods and algorithms for handling new sources of data is paramount.

### Social Media as a Surveillance Tool

As social media becomes more prevalent in everyday use for certain demographics, scientists are harvesting this data for analysing trends in a population, market research, sentiment analysis, and surveillance for public health and anti-terrorism purposes. The micro-blogging service, Twitter.com presents a promising data source for Internet-based surveillance because of the message volume, frequency, and public availability [25]. With approximately 332 million active uses as of January, 2016, Twitter contains a large volume of data that can be used for surveillance purposes. There is a growing body of research that utilizes this freely available information in order to correlate or predict twitter messages with specific public health outcomes (e.g. [28–31]). In Culotta [25], they used several regression models to predict actual influenza-like illnesses observed in a population to specific terms in twitter messages. In terms of surveillance for zoonoses, Twitter can be used to analyze keywords in messages to:

- Determine the public’s risk perception of a disease
- Identify new or unusual individual cases or emergent outbreaks
- Contribute to situational awareness of global AI patterns

Many studies that analyze Twitter data focus on the content of the messages and deciphering the meaning or associating it with a specific event, however, another challenging aspect of Twitter data is associating each message with geographic information. Tweets can contain geographic information if enabled by the user; it is estimated that only 2–5% of all Tweets are geocoded. Inferences can be made based on a user’s profile or content of the message to discern location, or in the case of local news reports that post to Twitter, from the publication’s origin or geographically explicit details contained in the full article.

Early warning systems are an essential goal of many surveillance systems (e.g. as examined in Hoye [18]review of 191 reports of surveillance in wild birds). The current system is in a large part, reactionary to new avian influenza viruses once they are detected in poultry, however, more upstream tracking of this information could provide important insights into public health messaging, relations between outbreak events, or further knowledge on the evolution and movement of these viruses [19]. Utilizing new technologies such as Twitter along with current surveillance systems may allow for near real-time risk surveillance of the information landscape associated with real-world outbreak events.

## Methods

### Data

#### Twitter Data

The public application-programming interface (API) of Twitter (i.e., Streaming API) was monitored for AI-related messages posted to the social media site during the period of September 26, 2015 to March 9, 2016. The global Twitter stream of messages was filtered based on a set of predefined keywords related to AI (Table 1). For each extracted message the date, text of the message, username, stated location of the user, and a fixed URL to the message was stored. Data were extracted on a continual basis from the streaming API using a custom Python script and the tweepy library (www.tweepy.org/). Data were added to a comma separated value database file on a nightly basis. Each day, a two-week time series plot and word cloud were generated to track recent patterns in messages throughout the study period which were automatically updated on an online repository. Messages that included geographic coordinates were identified and stored separately in a database file for mapping.

**Table 1:** Keywords used to search for AI-related Tweets

Keywords Used to Identify AI-related Tweets	'bird flu', 'avian influenza', 'avian flu', ‘poultry disease'

#### OIE Data

OIE reports were aggregated from the World Animal Health Information Database (WAHIS) Interface. Report data from the Avian Influenza for the years 2015 and 2016 were obtained, and truncated to match the study period based on the date the report was posted to the system. A number of items were tracked for the OIE data including type of report, virus type, number of cases and susceptible birds, whether wild or domestic birds were reported, and the start date of the outbreak. Location data were examined for veracity, but determined to be too imprecise for further analysis.

### Data Analysis Overall Methodology

**Fig 1:**
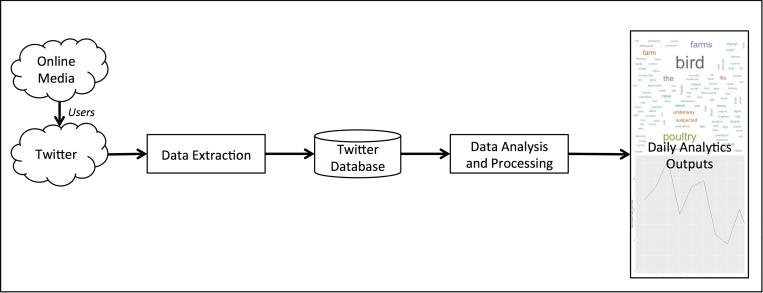
Data acquisition and processing pipeline

We developed a complete data processing and analysis pipeline in order structure and analyze the Twitter database. Our aim was to produce an online-capable set of methods that analyzed data as it arrived, rather than a purely retrospective analysis of the dataset. This was done in order to reflect a more realistic surveillance and/or situational awareness use-case for monitoring online content for disease-related information. A schematic view of the processing and analysis pipeline is presented in Fig 1. During the study period, daily outputs included a 2-week time series graph, a 2-week wordcloud, and a full-time series graph.

**Fig 2:**
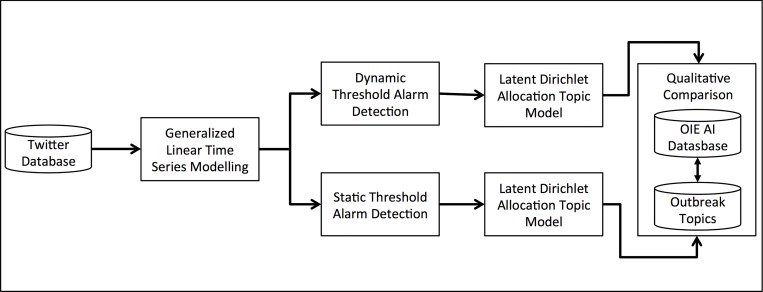
Data modelling, outbreak detection, and natural language processing pipeline

As can be seen in Fig 1, we are aiming to learn about online media by exploiting Twitter as a tool to find and quantify online media discussions related to AI. As messages by users (both human and machine), a sample of these will be noise (i.e., not related to AI in a substantial way). A statistical modelling approach was used to limit noise in the data and identify time periods of interest. The second stage of analysis pipeline was therefore modelling normal patterns of Twitter activity and identifying unusual time periods when there was a high-level of AI-related messages posted to Twitter (See Fig 2).

Once ‘outbreak’ periods were identified, the Twitter messages for these time periods were used in topic models that uncovered the key topics in messages during these times. Allowing the number of topics discovered to vary accommodated the possibility of multiple concurrent AI-related events. The output of the topic modelling was then compared to the OIE curated database to examine the degree to which detected AI-events on Twitter aligned or overlapped with events in the OIE database.

#### Modelling Twitter Activity

Twitter messages related to AI were aggregated by day, and a daily time series corresponding to the count of messages was created. The temporal pattern in the time series of AI-related tweets was used to identify ‘outbreaks’ or unusually high days or sequences of days in terms of the number of AI-related messages. In order to evaluate the temporal structure in the time series, we computed summary statistics, moving averages, and the autocorrelation function [32] (Box and Jenkins 1976). The ACF quantifies the degree of correlation of a time series with itself at different temporal lags.

Linear modelling of the time series was conducted using recently developed integer-valued autoregressive conditional heteroscedasticity (INARCH) models [33,34]). The INARCH framework provides a time-series model within a generalized linear modelling approach, including supporting different link functions, and incorporating effects of covariates. Our data consist of a relatively short, noisy time series (n = 147) of daily counts of AI-related Tweets. The INARCH model of the general form

$$g(\lambda_{t})=\beta_{0}+\sum_{k=1}^{p}\beta_{k}\tilde{g}(Y_{t-i_{k}})+\sum_{l=1}^{q}\alpha_{l}g(\lambda_{t-j_{l}})$$ (1)

which is a mixture of regressions on past observations with parameter weights *β*_*k*_ for each order of past observation up to P, and a regression on the past process mean governed by parameter *α*_*l*_. In our case, since we have only a short time series, we focus exclusively on the first term. Optionally, we can include covariates within this framework. A natural probability model for count data is the Poisson; as such we modelled the daily Poisson rate *λ*_*t*_, conditional on the mean of a subset of past observations. The Poisson model has the strong assumption of equality of mean and variance, and given the short and noisy time series we have, we also tested a negative binomial likelihood for the counts, which includes a dispersion parameter for extra-Poisson variation.

Model assessment was performed by techniques including minimizing the AIC, evaluating the normalized probability integral transform plot, and assessing forecast scoring rules as described in documentation for R package tscount which implements INARCH models [35]. Scoring metrics aim to quantify both the predictive distribution and the ‘sharpness’–how peaky the probability mass is in the predictive distribution (e.g., a highly sharp prediction is better than a flat prediction, yet this is lost when only using the point estimate prediction). The ranked probability score (RPS) and the mean logarithmic scores were used to compare the Poisson and negative binomial models [36,37]. The fitted model provides step-ahead predictions of daily Twitter message counts. We used the model fitted values as inputs into the next stage of the data analysis pipeline, detecting ‘outbreaks’.

Surveillance algorithms for univariate time series data are well developed [16]. We employed a simple cumulative sum (cusum) algorithm to detect outbreaks in an online setting. A cusum statistic is based on a cumulative summation of the deviation between the observed and the expected values of a process, given some thresholds for slack around the difference (i.e., the threshold for when exceedence is accumulated in the statistics) which is denoted *k* and a threshold for when the total cumulative deviance triggers an alarm and the statistics is reset, denoted *h*. Values for parameters *k* and *h* can be set based on the average run length during the ‘in-control’ state and the average run length in the ‘out-of-control’ state (detection delay) (See Rossi [38]) for a discussion of these parameters). We set the average run length to be 365 days in control, and 7 days out of control. The settings for the ARL were made in order to more heavily favour reducing false alarms vs early detection at the risk of a detection delay. An in-control ARL of 365 would be a full year of data before we could expect a false alarm, whereas an out-of-control ARL of 7 means we could ‘miss’ a shift in the process for up to seven days. Given the noisy nature of the data source being analyzed, conservative choices for ARLs were made to offset too many outbreak detections. The cusum framework provides a dynamic alarm threshold derived from the statistical modelling above. We compared this to a simpler alarm threshold, a fixed 95% confidence interval for the global process mean. Using the days that were denoted as ‘outbreak’ days, Tweets for these days were extracted and used for further natural language processing.

#### Natural Language Processing of AI-Related Twitter Messages

Latent Dirichlet Allocation (LDA) is a class of Bayesian mixture models that aim to discover topics from text data organized as a term-document matrix. LDA is an instance of a hierarchical multinomial model that seeks to generate (rather than classify according to known classes) characteristic topics from a set of text documents. In our case, each Tweet message is a document, and the text making up each message are the terms. The matrix representation is composed of columns, which are documents, and rows, which are words. The intuition behind this LDA model is that similar terms will be used for similar topics.

The LDA approach is effective at discovering topics from text data, and has become one of the most widely used approaches to unsupervised learning [39]. However, in the case of surveillance of social media activity for AI events, there are limitations with this naive approach. Firstly, the LDA models assume a static corpus of text data that is mined at a single point in time, however text-data from the web (whether from social media or web-scraping) is constantly evolving over time. This is especially true in the case of online surveillance. The notion of a static database of data extracted from the web is of limited utility when data are continually being updated. A second limitation of LDA is that the temporal ordering of documents is ignored in classical LDA. Temporal algorithms incorporate the timestamp of each message that factor in the temporal dependence in events giving rise to AI-related events. A final limitation in the context of social media messages is dependence between messages sent by the same author. The author-topic model includes dependence between messages while discovering topics [40].

The statistical modelling and outbreak detection algorithms therefore served to overcome several of the limitations of applying classical LDA. In order to process the data for analysis of the content of Twitter messages using LDA, several data cleaning operations were performed on the data (see Table 2).

**Table 2:** Data cleaning operations performed on Tweets prior to Latent DIrichlet Allocation topic modelling

Operation	Rationale
Lower case	Needed in order to ignore difference in text based only on case
Remove ‘RT’	Facilitates identifying duplicate entries
Remove usernames	Not related to AI content
Remove links	Not related to AI content
Remove punctuation	Not related to AI content
Remove leading and trailing spaces	Not related to AI content
Remove duplicates	The quantity of tweets is already captured in the statistical modelling, so duplicates not necessary for topic discovery
Remove stopwords	To focus NLP analysis on meaningful text
Stem words	To facilitate comparison of text data
Remove original search terms	Since only a five search terms were used, the differences in match frequency between difference messages was deemed not relevant so these were removed

#### Comparing Signals in Social Media to Official Surveillance Data

The time series of discovered topics was compared to the timeline of AI events as recorded in the OIE reports. We qualitatively compared these datasets in order to see to what extent patterns in the social media data analysis could be linked to events in the surveillance reports. Our objective in this synthesis was to identify both the nature of concordant patterns; where social media analysis aligned with surveillance data, as well as discordant patterns; where events occurred in the social media stream that were not part of official surveillance data, and vice versa. In so doing, we aimed to identify biases and characteristic in social media data relative to the OIE data. Data and code to implement the methods described here are available at https://github.com/colinr23/AITweets.

## Results

### Overall patterns in social media and official surveillance data

Over the course of the study period, a total of 38,191 Tweets were obtained related that matched at least one of the keywords in Table 1. The vast majority (n = 36,985) of messages matched one keyword, 1184 matched two, and 22 matched three keywords. These messages were contributed by 15,965 unique users, over the period from September 26, 2015 to March 9, 2016. Geo-location data (i.e., latitude and longitude coordinates) were obtained for only 36 messages, so were not analyzed in detail. General location information obtained from the user profile was obtained for most observations, which included 9061 unique text descriptions of locations to varying level of detail (See Table 3).

**Table 3:** Sample of location information available from profile information for AI-related Twitter messages

Ten Randomly Selected Location Entries in Tweet Database	"Chicago", "Nellore, andhra pradesh, India”, "Geneva, Switzerland”, "American Farms & Ag-Busineses", "Western Canada info@farms.com", "Toronto, ON", "Nigeria" "Hong Kong", "Auckland, New Zealand", "USA, Phoenix, AZ"

**Fig 3:**
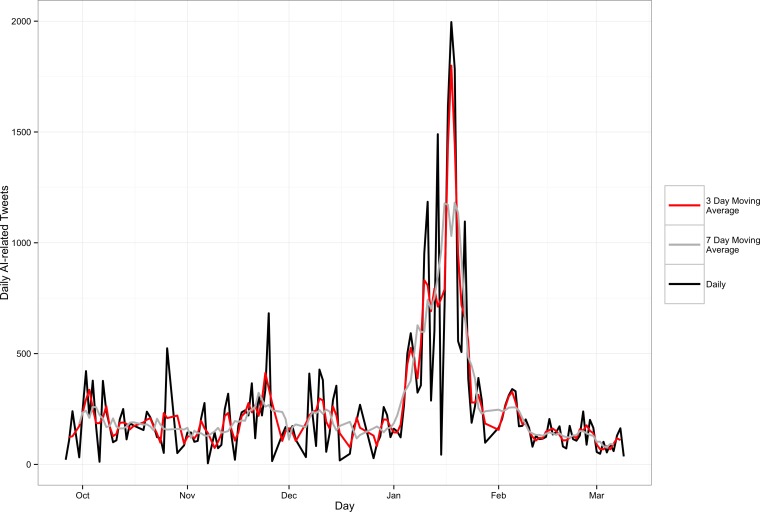
Time Series of AI-related Tweets including the raw count data (black), and three (red) and seven (grey) day moving averages.

The temporal pattern in observations is given in Fig 3, which shows the daily count of AI-related Tweets during the study period and two moving averages. At several points during the study period, the script harvesting Tweets from the API was interrupted and had to be restarted, which may have caused very low values where they would not have otherwise been observed. The moving averages account for these interruptions, however in our modelling we employ the raw count data because this is the nature of the data collection methodology. The script was never inactive for more than one day, so the overall temporal trends are reflective of the true distribution of activity on Twitter related to AI. The most marked observation from Fig 3 is a huge spike in activity in mid-January. Secondary spikes in activity are evident in late October, late November and perhaps early October. Part of the objective of the analysis that follows is to better understand the information content embedded in these patterns, and to assess the surveillance-value of social media for AI.

**Fig 4:**
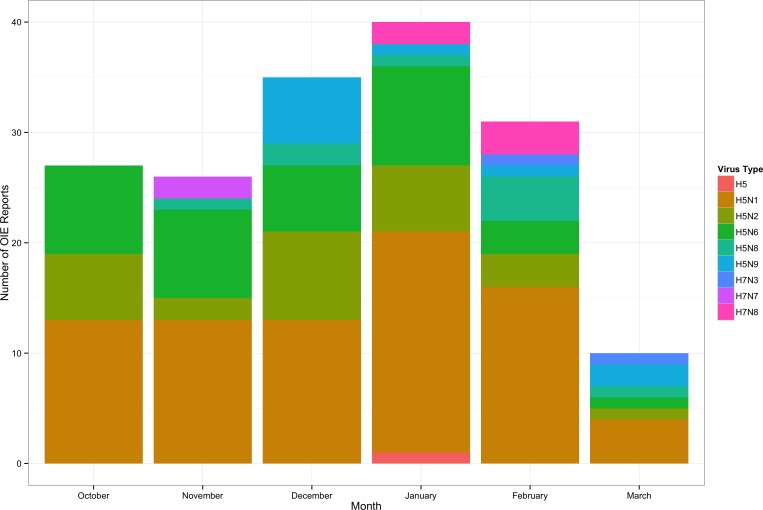
Time series of AI reports provided to the OIE during the study period.

OIE data extracted for the coincident time periods are given in Fig 4, giving the total number of reports by month and the relative distribution of AI virus types described in the reports. Qualitatively, the distributions look similar, with the highest number of reports in January. In December, several spikes in the Twitter data at the daily scale indicate a lot of activity during this month as well, and in the OIE data December was the second highest reporting month. To investigate further, we enumerated the month-to-month correlation between the two datasets, finding a Pearson’s correlation coefficient of 0.746, indicating a strong positive association between the monthly observations. However, given the low sample size, we cannot place much confidence in this finding. The difference in magnitude precludes direct comparison at a more granular temporal scale.

### Statistical modelling and outbreak detection in social media data

The raw time series data presented in Fig 3 were used as input into regression models for identifying normal patterns and one-step-ahead predictions in AI-related Twitter activity. Recall from Fig 1 that our purpose is to use Twitter activity as a proxy for online media in general; utilizing the ‘crowd’ on Twitter to curate and identify important events related to AI online.

The autocorrelation function showed a strong correlation structure in the data. The highest correlation was with the day previous, and strong correlations up to a five-day lag, after which it starts to decline (with a spike at 8 days). This temporal structure is an important characteristic of the data in order to take a time series modelling approach. Also, this is expected given that we expect AI events to persist over several days and related online activity to follow this pattern. The five-day correlation structure was used to help parameterize the INARCH model, which we specified to regress each day’s count on the previous five-days of counts in the time series. As discussed earlier, two models were fit to the data: a Poisson model and a Negative Binomial model that incorporates higher variance in the counts. The higher variance was also important given the data collection gaps in the time series that may have impacted the temporal structure of the observations.

The model coefficient estimates and assessment statistics are presented in Table 4 and Table 5. The beta coefficients can be interpreted as the effect of the n^th^ previous day on the current days count of AI-related Twitter messages, where n = 1,2,3 for *β*_1_,*β*_2_,*β*_3_ and so on. The estimates of each of the past observations on the count were very similar across the two models. However the model statistics demonstrated a much better fit for the negative binomial model that included the dispersion parameter (σ^2^ in Table 5), indicated by a lower AIC, higher log-likelihood, and lower values of the logarithmic and ranked probability scores [37].

**Table 4:** Results of Poisson time series regression INARCH model for daily time series of AI-related Twitter activity (Log-likelihood: -8907.42, AIC: 17826.85, log score: 6.26, ranked probability score: 78.76)

Parameter	Estimate	Standard Error
*β*_0_	36.49	2.05
*β*_1_	0.39	0.008
*β*_2_	<0.00	0.008
*β*_3_	0.30	0.008
*β*_4_	0.057	0.008
*β*_5_	0.10	0.007

**Table 5:** Results of negative binomial time series regression INARCH model for daily time series of AI-related Twitter activity (Log-likelihood: -920.29, AIC: 1854.58, log score: 60.59, ranked probability score: 109.36)

Parameter	Estimate	Standard Error
*β*_0_	36.49	28.70
*β*_1_	0.39	0.120
*β*_2_	<0.00	0.102
*β*_3_	0.30	0.113
*β*_4_	0.057	0.111
*β*_5_	0.10	0.103
σ^2^	0.433	NA

**Fig 5:**
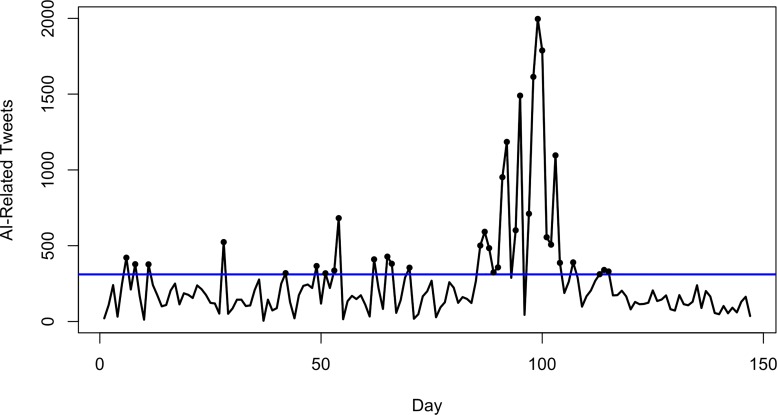
Observed daily time series of AI-related Twitter activity, black circles indicate significant errors (possible outbreaks) based on the static threshold criterion (blue line).

The probability integral transform histogram also indicated a more even distribution for the negative binomial model (not shown), which suggest a sharper forecast distribution. We therefore used the negative binomial model as the basis for generating expected counts of AI activity. The model values and the actual values are presented in Fig 5. We can see that the predicted values often lag the actual values that is a common characteristic of time series regression models, however, the normal and abnormal activity is apparent. Also note that the model values are prospective in nature, so prediction are based only on previous observations up to a temporal lag of five days (as determined by the ACF plot above).

**Fig 6:**
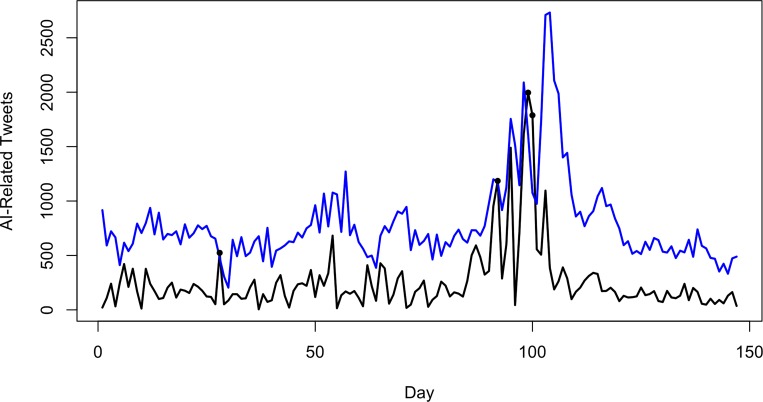
Observed daily time series of AI-related Twitter activity, black circles indicate significant errors (possible outbreaks) based on the dynamic threshold criterion (blue line).

Two methods were used for outbreak detection. A static threshold (denoted by the horizontal line in Fig 5) was determined based on the 95% confidence interval for the process mean. In Fig 5 days that exceed this threshold are denoted with a dark circle. In total, there were 34 days that were identified as anomalous using this method. The cusum algorithm was much more conservative in nature, identifying only 4 days that were unexpected (Fig 6).

### Natural language processing of AI-related Twitter messages

**Fig 7:**
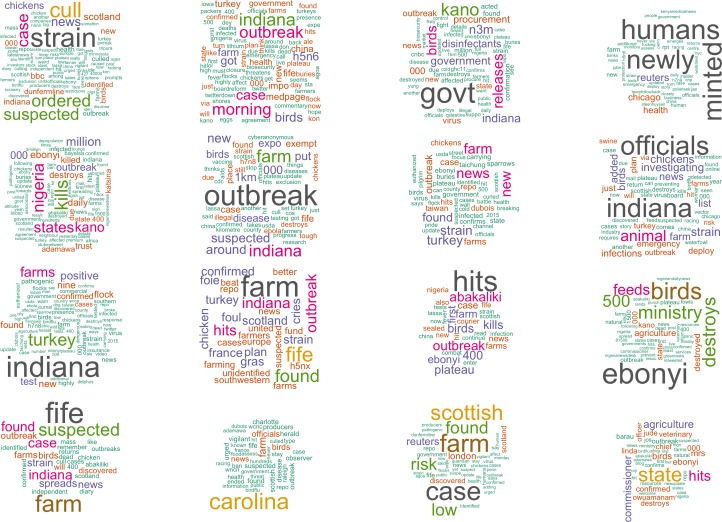
Wordclouds generated from original Tweets associated with topic models discovered from outbreaks based on the static threshold method (only 16 topics of 31 shown here).

**Fig 8:**
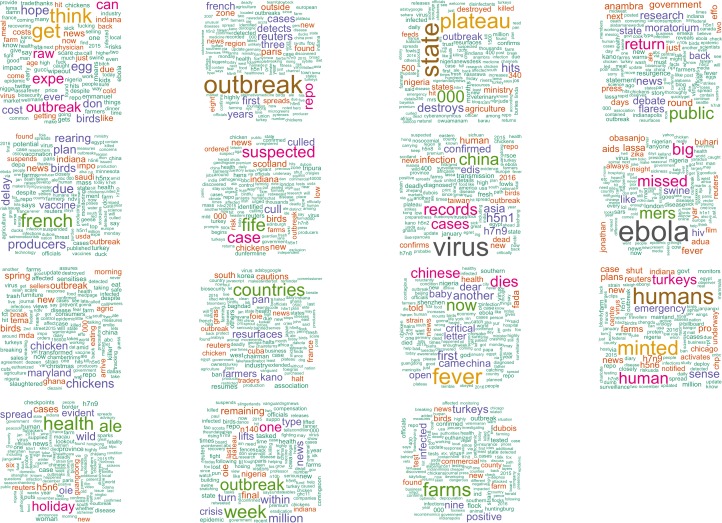
Wordclouds generated from original Tweets associated with topic models discovered from outbreaks based on the dynamic threshold method.

The days identified by the static (Fig 5) and dynamic (Fig 6) outbreak detection algorithms were investigated to determine the nature of the topics and locations (Table 6) embedded in the social media data (as indicated in Fig 2). The cross-validation step to determine the number of topics in the data found the optimal number of topics for the static data to be 31, and for the dynamic data to be 15. A set of wordclouds for the 16 topics from the static method is presented in Fig 7, and all 15 topics of the dynamic method in Fig 8.

**Table 6:** Top ranking locations based on profile of account for static and dynamic outbreak AI-related Tweets.

Location Rank—Static	Count	Location Rank—Dynamic	Count
Nigeria	1220	Nigeria	373
Global	979	Lagos, Nigeria	241
Lagos, Nigeria	807	Global	153
Lagos	469	Lagos	122
USA	434	United States	100
United States	363	USA	91
Mumbai, Maharashtra	212	London	53
Worldwide	196	Abuja, Nigeria	51
Abuja	186	Worldwide	46
London	181	Abuja	43
Abuja, Nigeria	161	Africa	40
New York	130	New York	39
India	129	United Kingdom	39
Africa	117	UK	38
UK	110	Canada	30
Philippines	105	lagos	29
Canada	94	Philippines	27
Earth	94	Scotland	26
LAGOS NIGERIA	94	NIGERIA	25
United Kingdom	89	Earth	22
Eastbourne, England	88	Indonesia	22
lagos	84	Lagos Nigeria	21
Washington, DC	80	Bonita Springs, FL	20
Delmarva	78	Germany / Europe	20
Lagos Nigeria	74	Eastbourne, England	19

Worth nothing in Fig 7 is the frequency of geographic place names, which indicate topics are associated with specific AI events reported in online media. We examined in detail of the topics discovered by the models, and interpreted the main theme or article associated with the topic. For some topics, they were linked to one specific story, whereas others included mixtures of articles around a common theme. Overall, 10 out of 31 topics were classified as unknown and ambiguous topics were discovered by the static method which included many more days and topics, whereas only one out of 15 was ambiguous for the ‘outbreak’ days identified by the dynamic detection method.

#### Comparing Signals in Social Media to Official Surveillance Data

**Fig 9:**
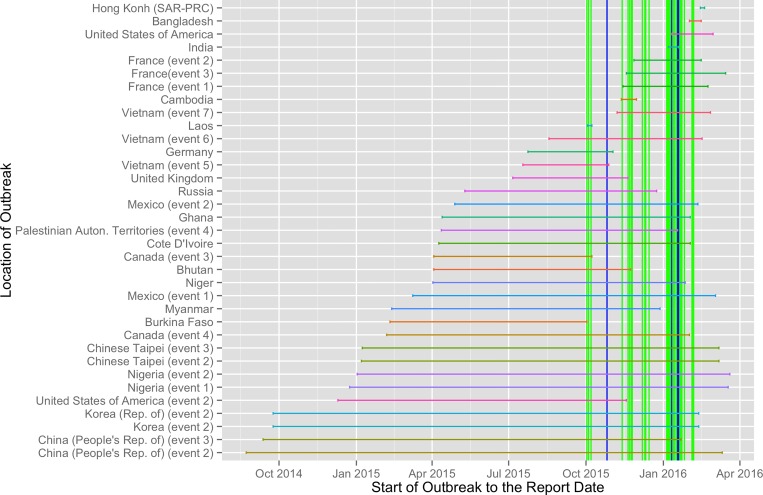
Official OIE outbreaks vs outbreaks identified on Twitter, blue vertical lines indicates dynamic method, green static method.

The curated OIE database which included only reports that contained the most recent data for current and completed outbreaks of AI during the study period contained a total of 73 observations. A comparison of the timing of these events relative to the outbreak events is given in Fig 9.

The Indiana outbreak event that according to OIE reports started on January 11^th^, 2016 included an outbreak of HPAI-H7N8, was first reported on in online media in the Twitter data on January 16^th^, 2016. The number of messages (after removal of duplicates) discussing the event was 119 on January 17^th^, an additional 212 on January 18^th^, 221 messages on January 19^th^, 324 on January 20^th^, and 31 and 29 the following days after that.

**Fig 10:**
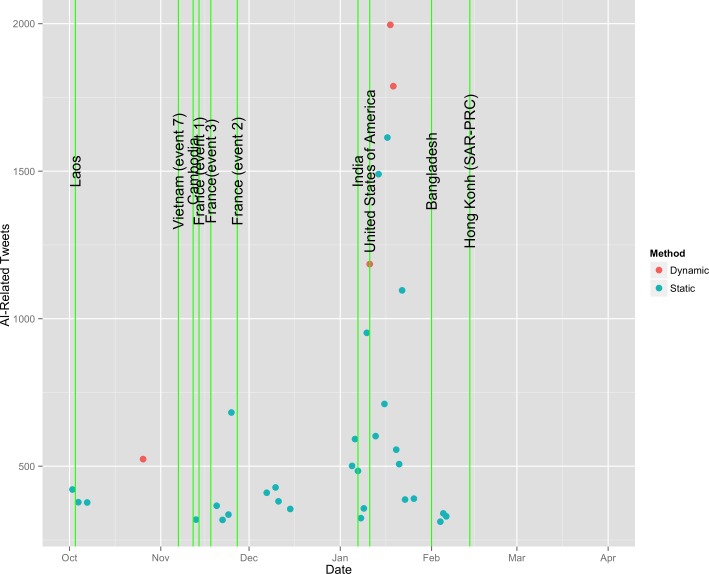
Official OIE outbreaks that started during the period of surveillance vs outbreaks identified on Twitter, green vertical lines indicate start dates obtained from OIE reports.

Investigating the dynamic outbreak in late October which does not seem to correspond to any new outbreaks based on Fig 1, we find 192 AI-related messages on that day. Table 7 reports the theme associated with messages on that day, finding that the vast majority (141/192) were related to a single article in Reuters titled ‘MERS, Ebola, bird flu: Science's big missed opportunities’. Other messages were related to general AI articles, the identification of a new outbreak in South Korea, and a suspected turkey shortage in advance of American Thanksgiving. Note also from Table 7 that only 11% of the messages were not substantively about AI. The outbreak was identified by the dynamic method because the article was posted during a time of relatively low activity, so the dramatic increase was flagged as unusual, although the response was not high enough to flag this using the static threshold. The increase was not due to a new outbreak explains its lack of concordance with any OIE reports during that time in Fig 10.

**Table 7:** Top ranking locations based on profile of account for static and dynamic outbreak AI-related Tweets on October 26^th^, 2016.

Theme	Number of Messages
Reuters Article	141
General AI	22
Newly Detected Outbreak in Korea	5
Thanksgiving Turkey Shortage	3
Specific AI	2
Azerbaijan Canada Taiwai	1 each
Not Related to AI	16

## Discussion

The analysis here provides a synoptic view of using social media as a tool for sensing online media related to AI. We have demonstrated a fully automated methodology to acquire, structure, model and analyze social media data related to AI at a global scale. This means that each process outlined in Fig 1 and Fig 2 (except for qualitative comparisons) are automated and computed online. Such a process could support a more comprehensive decision support tool or dashboard for monitoring social media AI trends and events. The method demonstrates that there may be surveillance value in understanding the media landscape related to AI outbreaks and responses to them across the globe. The modelling analysis revealed that in general, time periods identified by the dynamic threshold algorithm identified more relevant topics and many linked to known AI events as reported in the OIE data. The topic models revealed that the AI events in Nigeria were the most important in the social media data, garnering the most amount of activity. During the study period, the outbreak with the highest numbers of cases were actually in Chinese Taipai, but there were relatively less messages related to these outbreaks compared to Nigeria or outbreaks in France or Indiana, USA. The People’s Republic of China has strict controls on use of social media, including banning Twitter, so this is not unexpected. Also, a limitation of our analyses was that it was constrained to English-language content only, so non-English speaking countries and/or countries with low Twitter penetration were undersampled.

From a methodological perspective, some potential limitations are worth noting. Firstly, while the outbreak detection framework used here (i.e., cusum analysis) provides for automated analysis, there is a danger in employing step-ahead model outputs as inputs to the outbreak detection in that the detected outbreaks are dependent on the fit of the model to the data. In essence, the model assessment step (here using AIC and forecast sharpness scores) is critical the success of the subsequent detection step in the analysis pipeline. In practice, this means that some care is required with baseline data to select appropriate model forms. A related issue is tuning the model estimation step to reduce over-fitting in the context of prospective real-time surveillance. Because we only ran the model on one season’s worth of data, we were not able to investigate this aspect in detail. However, prospective surveillance algorithms typically utilize some combination of historical and current data in estimating baseline values [41], and such an approach could be adopted here when longer-term data are available. To further explore these issues in detail, additional work on surveillance modelling and event detection in social media data streams is needed.

In terms of effectiveness of Twitter for AI-risk surveillance in North America, the platform can provide timely and current information on trends and real-world events related to AI. One of the challenges to this form of media surveillance is understanding the degree to which these trends and events related to surveillance objectives. Similar studies of web-based surveillance for human disease such as Google Flu Trends has demonstrated shortcomings in outbreak detection capabilities due to conflation of search activity for flu *information* with search activity relating to new *infections* [27]. Models for disentangling search data for information from those for new infections have recently been demonstrated by Broniatowski [7]. In our analyses, the vast majority of AI-related messages were what might be termed ‘awareness’ oriented, aimed at publicizing and communicating AI-related information. As is the case with influenza, such information may have significant value for public and animal health planning. For example, Smith et al. 2016 focus on influenza awareness with Twitter showing how these messages differed when compared to outbreak-oriented message distributions. Awareness data distributions tended to be spikier and more short-lived than infections message distributions. The degree to which social-media-driven contagion of health related information can induce public fear has also recently been demonstrated in the context of the ebola outbreak in West Africa [42,43] Modelling to understand the spread of health-related information is an important part of evaluating public perceptions and developing sound risk communication protocols.

The qualitative comparison of OIE reports and social media data suggest that the social media stream provides a timely filter of online content for new AI events. The time delay between the start of the outbreak in Indiana and its reporting and detection online was five days. Developing this degree of timeliness in detection at a global scale could provide an effective tool for filtering the information landscape and developing visualizations that allow one to query, track, and identify events across the global disease landscape. However the detection and reporting in the Indiana outbreak may differ significantly from other jurisdictions.

Examining the content and themes of messages revealed that most were related to real and reported AI-events. Twitter acted as an efficient tool to access reports of AI events in news articles, blog posts, and other sources of online media. While the topic models in this analysis only analyzed Tweet content, which tend to be article titles, there may be value in extending the analysis deeper by crawling to linked full articles and using similar natural language processing models on the full content of the articles. While we focused this analysis on dominant themes and events, some of the hidden value in social media data is embedded within less frequently posted (e.g., one user posted ‘Had a great day at the NC State Fair. Best: The Jaycee Turkey Shoot. Worst: No chicken exhibits due to bird flu concerns’). Developing a system capable of identifying important themes as well as interesting messages that are standalone would provide both broad thematic and granular information on an ongoing and real-time basis.
